# Glycoconjugates: What It Would Take To Master These Well-Known yet Little-Understood Immunogens for Vaccine Development

**DOI:** 10.1128/mSphere.00520-19

**Published:** 2019-09-25

**Authors:** Fikri Avci, Francesco Berti, Peter Dull, John Hennessey, Viliam Pavliak, A. Krishna Prasad, Willie Vann, Michael Wacker, Olivier Marcq

**Affiliations:** a Department of Biochemistry and Molecular Biology, Center for Molecular Medicine and Complex Carbohydrate Research Center, University of Georgia, Athens, Georgia, USA; b GSK Vaccines, Siena, Italy; c Bill and Melinda Gates Foundation, Seattle, Washington, USA; d Independent Researcher, Lower Gwynedd, Pennsylvania, USA; e International Vaccines Institute (IVI), Seoul, Republic of Korea; f Citranvi LLC, Chapel Hill, North Carolina, USA; g FDA-CBER, Silver Spring, Maryland, USA; h Wacker Biotech Consulting, Thalwil, Switzerland; i Sutrovax, Foster City, California, USA; University of Missouri-Kansas City School of Medicine

**Keywords:** T-cell immunity, capsular polysaccharide, carrier protein, clinical trials, glycoconjugates, immunity, immunology, infectious disease, synthetic drug carriers, vaccines

## Abstract

Glycoconjugate vaccines are a critical component of the medical arsenal against infectious diseases. This established field continues, however, to experience failures in the clinic. The lack of fundamental understanding of factors controlling clinical efficacy of glycoconjugate vaccines is discussed while key parameters demanding focused and collaborative research are identified.

## PERSPECTIVE

The tremendous impact of vaccines on human health over the last century is indisputable. The more recent contributions from glycoconjugate vaccines have been impressive, with rapid impact on diseases caused by a number of encapsulated bacteria, including Haemophilus influenzae type b, pneumococcus, and meningococcus. Many additional pathogen targets are also under active investigation (1–7), while the increasing incidence of antibiotic resistance might prompt the need to develop vaccines for diseases treated so far by therapeutic antibiotics. The impressive track record of safety and efficacy of the many licensed prophylactic products might lead us to assume that glycoconjugate vaccine design is fundamentally understood. However, this is not the case, as development of new glycoconjugate vaccines continues to be hampered by unexpected technical and medical issues during clinical development. We postulate that such issues can be alleviated by a greater basic understanding of how glycoconjugates operate. We further argue that this can largely be tackled by concerted and collaborative efforts across academic, government, nongovernment, and industry partners to gain understanding of glycoconjugate design and production and for optimal conjugates to reach their target population.

The lack of public or private funding to support freely disseminated basic translational research in understanding critical vaccine attributes complicates advances in the field. Expert discussions that led to the core content of this article highlight both the need for and the usefulness of exchanges across the field.

While glycoconjugate vaccines have provided great health benefits, the design/construction of such vaccine has often been empirical, with production processes prone to deviations, incompletely characterized glycoconjugate antigens, and vaccine products with limited immunologic evaluations. Especially problematic has been the inadequate understanding of the mechanisms by which the human immune system, both naive and previously exposed to the target pathogens, interacts with these complex antigens. For glycoconjugate vaccines to fulfill their full public health potential, experienced academics, regulators, and product developers need to pool the best practices learned and the knowledge accumulated over the past 40+ years of work in an open and transparent manner. With the identification and communication of critical research gaps, funders will then be better able to step in and new product developers can avoid mistakes of the past, allowing for lower-cost and more efficient development pathways to new products. This work attempts to identify specific areas of focus and highlight and prioritize gaps in the glycoconjugate vaccine development arena.

## PRODUCT CHARACTERISTICS THAT INFLUENCE IMMUNE RESPONSE

Many variables in the design, development, and production of glycoconjugate vaccines influence their immunogenicity and presumably their efficacy. The choice of saccharide size, carrier protein, conjugation chemistry, and formulation are some of the key decisions faced in every glycoconjugate development program (e.g., see references 8 and 9). Several of these factors have been proposed in the literature to explain why a given glycoconjugate vaccine or a regimen of such vaccines underperformed in clinical studies (e.g., see references 10 and 11). New glycoconjugate vaccines are developed based mostly on company-specific design, know-how, expertise, and technology platforms, typically without benefit of solutions derived from the broader community experience. Moreover, introduction of new technologies arises sometimes from a need for product differentiation to create a competitive advantage or to navigate a complex intellectual property landscape. The result is unexpected failures or inferiority of some new glycoconjugate vaccines in clinical trials and a limited number of successful, reliable glycoconjugate production technology platforms. As a consequence, confidence around the maintenance of product safety and efficacy for regulatory authorities and manufacturers relies closely on manufacturing process control and clinical and commercial product characterization without a clear understanding of the critical quality attributes of the products being made.

Despite the commendable focus of academic and industry laboratories to bring forward new innovative or follow-on vaccine candidates as quickly as possible, the heart of the issue is the lack of available data to support a community-wide basic understanding of the critical design features and attributes that predict optimal protective immune responses in the relevant populations. This challenge is further complicated due to the nonavailability of reliable animal models that can correlate and predict clinical efficacy. Due to limited public information from the many previous successful and unsuccessful glycoconjugate vaccine programs as well as current active endeavors in the private sector, the optimal product characteristics of and immunological responses to glycoconjugate vaccines are unclear and will require significant efforts to define. We suggest below specific areas of focus for these collaborative efforts.

**FIG 1:**
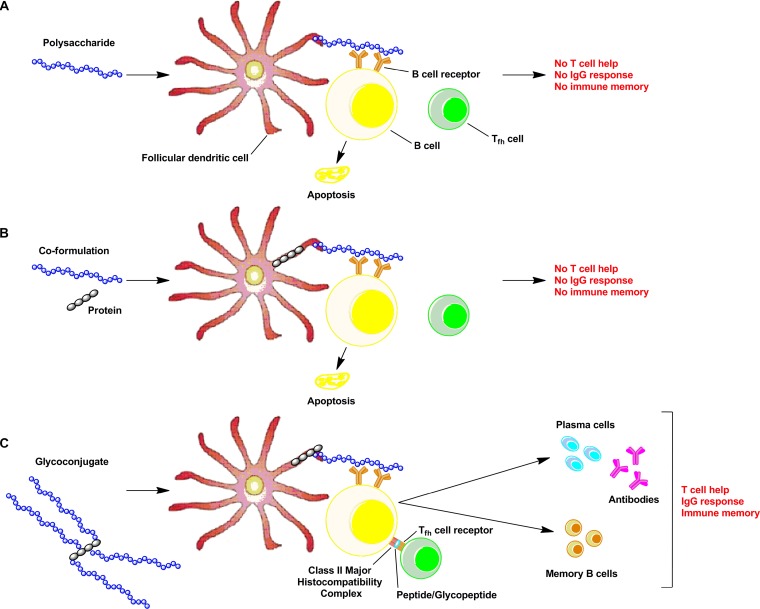
Schematic representation of the interactions of polysaccharide (A), polysaccharide and protein coformulation (B), and glycoconjugate (C) with FDC, B cells, and Tfh cells and the associated immune response. For polysaccharide and polysaccharide and protein coformulation, no IgG response and immune memory are induced because of the absence of T cell help. For glycoconjugate, the loading of peptide or glycopeptide into MHC and the engagement of TCR elicits an IgG response and immune memory.

**(i) Elucidation of the immune response mechanisms induced by glycoconjugate vaccines.** While glycoconjugate vaccines have provided great health benefits in controlling bacterial diseases, their chemical conjugations have often been empirically driven, with variably controlled production processes (e.g., conjugation) and analytical profiles, resulting in variably immunogenic glycoconjugate vaccine molecules. Process and quality consistency impact the composition of the vaccine product and in turn influence the immunogenicity and efficacy of glycoconjugate vaccines. One critical factor for enhancing glycoconjugate vaccine immunogenicity is our understanding of how glycoconjugate vaccines induce adaptive immune responses. While the traditional hypothesis suggests a peptide presentation to helper T cells (2, 12), a new model proposes the presence of carbohydrate-specific T cells (i.e., Tcarbs) and their function in inducing adaptive immune responses in glycoconjugate immunization (13–16). The hypothesis that led to the discovery of this new model was that carbohydrate antigens in their pure form do not bind to major histocompatibility complex class II (MHCII) and therefore are not presented effectively to T cells (Fig. 1A). However, when conjugated with a carrier protein, a peptide-bound, processed carbohydrate epitope (glycan_p_-peptide) is generated in the endolysosomes of antigen-presenting cells (APCs). Through binding of the peptide portion to MHCII, the carbohydrate portion (glycan_p_) is presented on the APC surface for T-cell recognition (Fig. 1C). Tcarb-mediated immune responses induced by glycoconjugate immunization have been demonstrated to yield protective immunity in controlled *in vivo* model systems through either depletion (13) or adoptive transfer (15) of epitope-specific CD4^+^ T-cell populations. Most recently, it was demonstrated that polysaccharide structure dictates mechanism of adaptive immune response to glycoconjugate vaccines (16). In that study, four clinically important glycoconjugate vaccines were tested for their mechanism of action. Three of the four glycoconjugate vaccines tested induced adaptive immune responses regulated by Tcarbs. However, the meningococcal group C (MenC) conjugate vaccine immunity was predominantly restricted to peptide-specific T cells. The explanation proposed for the lack of Tcarb stimulation by MenC conjugate was that MenC polysaccharide is substantially depolymerized in the endolysosomes, yielding small oligosaccharides (as small as a monosaccharide) that do not sufficiently mask the peptide in the MHCII binding groove and therefore do not elicit Tcarb responses. The relative contribution of peptide or glycan presentation models for glycoconjugate vaccine efficacy is yet to be fully explored. The current understanding based on the recent literature is that the glycan presentation is a natural result of the difficulty in cleaving the covalent/synthetic bond established between the glycan and the carrier protein and the slow or partial processing of the polysaccharide in the endolysosomes of antigen presentation cells. Thus, peptide-bound processed glycan epitopes are formed during processing in the endolysosomes to then be presented to helper T cells. To further expand the understanding of this mechanism of action, it would be important to characterize the presence of Tcarb in a definitive way by isolating more Tcarbs and by determining the structure of the Tcarb bound to a glycopeptide by X-ray diffraction (17). The studies described above lay the groundwork for future investigations pertaining to elucidation of structural requirements for MHCII-dependent carbohydrate presentation; elucidation of molecular interactions yielding T-cell stimulation by epitopes generated from processing of glycoconjugate vaccines; and design and synthesis of structurally defined, knowledge-based, protective new-generation glycoconjugate vaccines. For existing products, glycoconjugate construction has been an empirically driven process of linking two molecules (carbohydrate and protein) without considering the molecular and cellular immune mechanisms critical for conjugate vaccine efficacy. A deeper understanding of these mechanisms (e.g., antigen uptake, processing, and T-cell activation) may also provide opportunities for optimizing novel chemical or biological conjugation technologies that have been recently described (18–20). Thus, delineating T-cell-mediated immune activation pathways by glycoconjugate vaccines has important implications for using these vaccines to control or eliminate infectious diseases globally.

**(ii) Assessment of the impact of polysaccharide size, structure, functionalization, and conformation on immune responses to the conjugated polysaccharide.** It is commonly accepted that oligosaccharide antigens with chain lengths longer than the minimal epitope length may behave as native polysaccharides. In certain cases, the protective epitope is nonlinear and the length of the polysaccharide sequences must be investigated for protective conformations in the context of the conjugate. This is further complicated by the need to define the role of labile side groups (e.g., O-acetyl, pyruvic acid, etc.) in the immunogenicity of the epitope. As an example, clinical evaluation of multivalent group B *Streptococcus* (GBS) conjugate vaccines has been ongoing for nearly 2 decades (21) but the optimal length for the GBS type III saccharide and the putative existence of a conformational epitope are still being debated (22). In the clinic, we observe successes with glycoconjugate vaccines made with both oligosaccharides and polysaccharides, and efficacy induced by one or both seems to be polysaccharide dependent. As mentioned above in section i, it was demonstrated that polysaccharide structure can influence the specific mechanism of adaptive immune response to glycoconjugate vaccines (16). As conformational epitopes might be expected to be found, the field would benefit from improved molecular modeling of large polysaccharides. Finally, it would be useful to understand whether the size of the polysaccharide or the size of the conjugate makes the biggest impact on immune response.

**(iii) Evaluation of immune mechanisms by which carrier proteins alter the immune response.** Multiple carrier proteins have successfully been employed in licensed infant glycoconjugate vaccines (e.g., CRM_197_, tetanus and diphtheria toxoid, outer membrane protein complex [OMPC], and nontypeable Haemophilus influenzae [NTHi] protein D). The optimal choice is unclear, although glycoconjugates using CRM_197_ and tetanus toxoid dominate the commercial markets. Additionally, immune response to nontraditional carriers based on conserved proteins (e.g., NTHi protein D) may also contribute to protection through their independent action as immunogens. We need to develop a better understanding of T-cell responses induced by carrier proteins and differences between carrier proteins. Indeed, assuming that presentation of carbohydrate attached to carrier protein peptide to T cells via uptake to MHCII is a systematic/universal process, it is likely that certain peptide fragments might be better than others. This requires discovering the carrier protein epitopes presented to and recognized by helper T cells from glycoconjugate processing in antigen-presenting cells. The principles of structural vaccinology highlighted by Bottomley et al. (23) should be applied to carrier protein design. Ultimately, carrier proteins should probably be designed *in silico* to achieve optimal T-cell presentation. Critically, confirmation in humans, and more specifically in the target human population (e.g., infants), is essential to avoid optimizing to a specific animal model that may not correlate. This points to the need for great care in anticipating whether observations in animal models can directly inform proof of concept (POC) in humans.

**(iv) Understanding carrier-induced epitope suppression.** The introduction of several infant vaccines simultaneously or in close sequence has raised concern that the repeated exposure to conjugates based on any one of these carrier proteins might interfere with the antipolysaccharide antigen response. This “carrier-induced epitope suppression” (CIES) is of greatest concern with multivalent conjugate vaccines that contain the same carrier protein but also has to be considered with separate vaccines that use the same carrier protein. Prior immunity against a carrier protein has been proposed to modulate the serologic response to injected antigens attached to the same carrier (24). CIES has been proposed as one of the possible mechanisms through which an interference occurs resulting in a less robust immunogenic response by one or more of the constituent vaccine products or serotypes in a multivalent vaccine. Schutze et al. (25) proposed that epitopic suppression is induced through the expansion of the clones specific for the carrier protein epitopes and results from intramolecular antigenic competition between hapten and carrier epitopes. Based on these findings, a regulatory role was proposed for B cells, where through their capacity to process and present antigen, they would exercise a strong influence on the selection of immune responses. However, critical appraisal of clinical trial data by Pöllabauer et al. (26) concludes that neither the carrier protein type nor dose adequately explains observed interference. In five clinical trials of Haemophilus influenzae type b capsular polysaccharide (PRP) conjugate vaccines, enhancement of anti-PRP serum IgG has been demonstrated after coadministration of monovalent MenC conjugate vaccine with tetanus toxoid carrier. The authors conclude that empirical observations do not fit well with CIES as the single underlying mechanism of interference. Additional factors that may be specifically attributable to the polysaccharide-antigen competition, rather than to carrier-induced interference, may contribute to the diminished immunogenic responses in certain instances. In light of the confounding factors, additional research is required to verify the impact of CIES and understand the associated basic immunological mechanisms that contribute to polysaccharide-antigen competition.

**(v) Assessment of the impact of conjugation platform on immune response.** An added level of complexity in comparing glycoconjugate constructs stems from the variety of conjugation platforms (e.g., conjugation chemistry, multiple attachment versus single-point attachment of carbohydrate, presence/absence of linker, or sites of attachment on the carrier protein). From a broad overview of polysaccharide-protein conjugate vaccines, it appears that extremes of saccharide activation (very high or very low) have produced unexpected results in conjugate immunogenicity. In relevant cases, the level of O-acetylation and/or sialylation must be assessed as it might be relevant to immunogenic epitope preservation and therefore guide the selection of conjugation methods (27, 28). Some chemistries lead to stability issues, while some polysaccharides are more amenable to conjugation by certain methods than others. Consistent presentation of carbohydrate epitopes is essential as shown with synthetic oligosaccharides (29), but the choice of conjugation chemistry is not obvious for a given polysaccharide-carrier protein combination. Additionally, there are few published data to systematically evaluate the impact of linkers and sites of attachments on carrier proteins on immune response to saccharide haptens. Finally, new linkers and chemistries raise concerns due to the potential creation of immunogenic neoepitopes which may complicate the regulatory approval pathway for such products. For the field to truly move toward a rational glycoconjugate design, we need to understand how details of antigen structure in the vaccine molecule influence immunogenicity in humans generally and ultimately in the target population for the specific vaccines.

Quality attributes such as saccharide size, degree of saccharide activation, constraints on modifications of saccharide structure, use of linkers, density of attachment on carrier protein, or size distribution of conjugate particles appear important to control. New platforms (18–20) that allow the more precise control of such parameters should be used to develop test molecules for use in *in vitro*, *in vivo*, and *ex vivo* models to explore mechanisms of immune response to glycoconjugate vaccines. We do not know whether the Tcarb clone repertoire is dependent on the conjugation platform. The availability of well-defined test molecules for immunological experiments will be critical to our systematic evaluation of the impact of these quality attributes on the immune response to the conjugated haptens.

**(vi) Development of a greater breadth of analytical tools to better characterize glycoconjugates.** Most conjugates are produced from large polysaccharides (average molecular weight more than 100 kDa), resulting in lattice-like conjugates (average molecular weight often more than 1,000 kDa), which limits how much can be learned with current analytical methodology. Addition of adjuvants, surfactants, and other excipients makes characterization and stability monitoring more complex. Yet, new analytical tools are revealing more about these complex entities. Tools for physicochemical analyses, such as high-field nuclear magnetic resonance (NMR), particle-size distribution analyses, and perhaps cryo-electron microscopy can reveal structural details of glycoconjugates. We need to apply high-performance physicochemical analyses, combined with cutting-edge immunological methods, to better understand the structure-function relationship for glycoconjugates to better inform fine-tuning of glycoconjugate design and production. If the use of a lattice glycoconjugate format is established to be immunologically superior to (e.g.) a single-point attachment glycoconjugate format for a given disease target, technologies enabling site-specific conjugation might be favored in order to enable in-depth characterization of the glycoconjugate vaccine and better consistency of manufacturing. Ultimately, the linkage of the results from these improved analytical tools to human immune response safety and efficacy is critical to advancing our understanding of the true critical quality attributes of these vaccines.

**(vii) Development of better, more relevant *in vivo* or *ex vivo* models for evaluation of glycoconjugate vaccines.** It is obvious that research and development of glycoconjugate vaccines rely on animal models to establish scientific proof of concept that the vaccines can provide protection against the target pathogens. The animal models are by necessity focused on immune response to the vaccine and/or protection from challenge with the target pathogen, often with definition of surrogate markers of protection. However, these studies are often of limited relevance to human disease conditions and are unreliably predictive of the responses of the target human populations. Pursuit of “humanized” animal or *in vitro* models as done in oncology with xenograft models or the use of *ex vivo* models (e.g., cellular models) for evaluation of antigen processing, presentation, and/or functional activity may be a useful addition to the current approaches. Regardless, testing in animals is an essential precursor to clinical trials, which are the first true opportunity to assess the immunogenicity/efficacy of the vaccine candidate in the target human population. When possible, early use of new or improved glycoconjugate vaccines in controlled human infection models (CHIMs), such as those deployed for pneumococcal organisms (carriage) and typhoid (invasive disease), can assist in early-stage gating of vaccine candidates, depending upon the final target population.

**(viii) Tailoring glycoconjugate vaccines for specific target populations.** We know that the immune systems of humans of different age groups are not functionally identical (30). The quality of the immune response to a given glycoconjugate vaccine is also dependent on the naive or primed exposure of the patient to the target pathogen. Considering this, we might increase efficacy by studying optimal dosing regimen and schedule or adjuvanting in clinical trials (31). In addition, we still do not fully understand whether the Tcarb clones or the carrier-specific T-cell clones are triggered by a given vaccine candidate and how these events vary depending on the age of the receiving population. This is only an example of fundamental understanding that we are currently lacking that limits our ability to truly optimize the efficacy of conjugate vaccines and tailor them to specific target populations.

**(ix) Development of lower-cost glycoconjugate vaccines.** Vaccines such as the pneumococcal conjugate vaccine, given as a multidose regimen to infants in nearly all countries, make up a disproportionate fraction of the vaccine procurement budget in many low-income countries. Although financing mechanisms through international alliances such as GAVI (www.gavi.org) and UNICEF (www.unicef.org) support the purchase of these vaccines in the poorest countries, introducing new vaccines makes difficult tradeoff decisions necessary. It is important that the cost of manufacturing is prioritized early in the development of new vaccines, and funders like the Bill & Melinda Gates Foundation are increasingly becoming involved to facilitate these early decisions as well as making knowledge gained in this process more freely available. New glycoconjugate vaccines (e.g., GBS and *Shigella*) and high-valency (serogroup coverage) versions of existing vaccines are likely in coming years, and better understanding of the underlying technology will help ensure that these can be made at affordable prices. This work would also include improved deliverability for low-resource settings, which could result in multidose vial presentations and optimizing around maximal temperature stability.

## CONCLUSION

At present, the lack of understanding of what parameters govern efficacy and safety of glycoconjugate vaccines can result in unexpected clinical outcomes, which necessitate revision of production processes and substantial delays in vaccine development and licensure due to the need to repeat clinical studies. This is especially true for prophylactic vaccine products targeted to infants and otherwise healthy populations. Consequently, although minor changes can be evaluated in later-stage clinical trials, demonstration of safety and immunogenicity in a phase I trial and avoiding major changes in the processes for making glycoconjugate vaccines can improve the efficiency of vaccine development and licensure. If we can move forward significantly in the areas mentioned in this article, we will be able to more efficiently design and test glycoconjugate vaccine candidates based on an improved foundation of knowledge about glycoconjugate vaccines and how they work. This in turn will reduce failures in clinical trials and accelerate the path to licensure and our ability to address unmet medical needs.

Finally, we recognize that the broad variety of basic research work needed would be better conducted in settings where open access to results of the work is guaranteed. There are a number of options (academic, government, or industry laboratories or consortia of two or more of these), but fundamental agreement on broad and nonexclusive knowledge sharing is essential. Establishing consortia among key partners to address fundamental questions, funder- or institution-mandated sharing of early clinical data with or without incentives to reveal new learning in the field, or pathogen-specific multipartner efforts led by government or nonprofit organizations could be far-reaching and game-changing avenues leading to faster development of efficacious glycoconjugate vaccines, particularly for new or emerging bacterial pathogens (NIH-NIAID-mandated sharing for intramural work is an example of institution-mandated knowledge sharing; current efforts funded by the Bill & Melinda Gates Foundation with partners including PATH, Biovac [South Africa], and a variety of other contributors to develop a novel GBS glycoconjugate vaccine are an example of multi-institution and international partnership).
